# Factors associated with ultrasound-aided detection of suboptimal fetal growth in a malaria-endemic area in Papua New Guinea

**DOI:** 10.1186/s12884-015-0511-6

**Published:** 2015-04-03

**Authors:** Holger Werner Unger, Maria Ome-Kaius, Stephan Karl, Dupain Singirok, Peter Siba, Jane Walker, Regina Alice Wangnapi, Ivo Mueller, Stephen John Rogerson

**Affiliations:** Papua New Guinea Institute of Medical Research (PNG IMR), 60, Goroka, Eastern Highlands Province 411 Papua New Guinea; Department of Medicine (Royal Melbourne Hospital), The University of Melbourne, Post Office Royal Melbourne Hospital, Parkville, VIC 3050 Melbourne, Australia; Walter and Eliza Hall Institute of Medical Research (WEHI), 1G Royal Parade, Parkville 3052, Melbourne, Australia; Royal Infirmary of Edinburgh, 51 Little France Crescent, Edinburgh, EH16 4SA UK; Barcelona Centre for International Health Research (CRESIB), Rosselo 132, 08036 Barcelona, Spain

**Keywords:** Malaria in pregnancy, Nutrition, Anaemia, Fetal growth restriction, Small for gestational age, Mid-upper arm circumference

## Abstract

**Background:**

Fetal growth restriction (FGR) is associated with increased infant mortality rates and ill-health in adulthood. Evaluation of fetal growth requires ultrasound. As a result, ultrasound-assisted evaluations of causes of FGR in malaria-endemic developing countries are rare. We aimed to determine factors associated with indicators of abnormal fetal growth in rural lowland Papua New Guinea (PNG).

**Methods:**

Weights and growth of 671 ultrasound-dated singleton pregnancies (<25 gestational weeks) were prospectively monitored using estimated fetal weights and birthweights. Maternal nutritional status and haemoglobin levels were assessed at enrolment, and participants were screened for malaria on several occasions. FGR was suspected upon detection of an estimated fetal weight or birthweight <10^th^ centile (small-for-gestational age) and/or low fetal weight gain, defined as a change in weight z-score in the first quartile. Factors associated with fetal weight and fetal weight gain were additionally assessed by evaluating differences in weight z-scores and change in weight z-scores. Log-binomial and linear mixed effect models were used to determine factors associated with indicators of FGR.

**Results:**

SGA and low weight gain were detected in 48.3% and 37.0% of pregnancies, respectively. Of participants, 13.8%, 21.2%, and 22.8% had a low mid-upper arm circumference (MUAC, <22 cms), short stature (<150 cms) and anaemia (haemoglobin <90 g/L) at first antenatal visit. 24.0% (161/671) of women had at least one malaria infection detected in peripheral blood. A low MUAC (adjusted risk ratio [aRR] 1.51, 95% CI 1.29, 1.76, *P* < 0.001), short stature (aRR 1.27, 95% CI 1.04, 1.55, *P* = 0.009), and anaemia (aRR 1.27, 95% CI 1.06, 1.51, *P* = 0.009) were associated with SGA, and a low body mass index was associated with low fetal weight gain (aRR 2.10, 95% CI 1.62, 2.71, *P* < 0.001). Additionally, recent receipt of intermittent preventive treatment in pregnancy was associated with increased weight z-scores, and anaemia with reduced change in weight z-scores. Malaria infection was associated with SGA on crude but not adjusted analyses (aRR 1.13, 95% CI 0.95, 1.34, *P* = 0.172).

**Conclusion:**

Macronutrient undernutrition and anaemia increased the risk of FGR. Antenatal nutritional interventions and malaria prevention could improve fetal growth in PNG.

## Background

Fetal growth restriction (FGR) is associated with growth faltering in early childhood, increases infant mortality rates, and predisposes to ill-health in adulthood [1-4]. The burden of FGR and its potential consequence, low birthweight (LBW, <2,500 g), is estimated highest in low-income countries (LIC), where maternal undernutrition, short stature, malaria, anaemia, and HIV are important risk factors and pregnancy rates are high [3,5-7].

Researching causes of FGR in LICs is challenging because ultrasound for pregnancy dating and fetal growth monitoring is not yet widely available. Alternative estimators of gestational age such as last menstrual period are frequently unreliable [8], and late presentation to antenatal clinic precludes dating based on early pregnancy biometric measurements [9]. A number of studies evaluated factors associated with measuring small-for-gestational-age (fetal size/weight <10^th^ centile of a given weight standard, SGA) [3], commonly used as an indicator of FGR in epidemiological studies, yet few used ultrasound to determine gestational age and to evaluate fetal growth in utero.

Sequestration of *Plasmodium falciparum*-infected erythrocytes in the placental intervillous space is an important cause of LBW, most frequently due to FGR [10]. A small number of ultrasound studies have evaluated the effect of malaria infection, and at times additional factors such as maternal nutritional status, on fetal growth. In Thailand and Brazil, *P. falciparum* and *P. vivax* infection in early pregnancy was associated with lower fetal biometric measurements [11,12], while in Tanzania, third trimester fetal growth alterations were observed following malaria infection before 20 weeks’ gestation [13]. Infections tended to have most impact on growth in late second and early third trimester in a Kenyan cohort [14]. In the Democratic Republic of Congo, a low mid-upper arm circumference (MUAC), short stature, malaria and anaemia were associated with SGA, [15], while in Thailand a low body mass index (BMI) was associated with reduced biparietal diameter z-scores [11]. In another Congolese cohort, undernourished women were at increased risk of SGA due to *P. falciparum* infection [16]. Overall, the number of ultrasound studies evaluating the role of undernutrition and malaria as causes of suboptimal fetal growth in LICs is limited, in particular outside of sub-Saharan Africa and South East Asia.

Antenatal care is a window of opportunity to improve fetal outcomes. Mapping principal causes of FGR in LICs provides a platform to develop, test and implement prenatal interventions to improve fetal growth and reduce LBW. We evaluated factors associated with FGR in fetuses of women co-enrolled in a randomised controlled trial evaluating intermittent preventive treatment of malaria in pregnancy (IPTp) in Papua New Guinea (PNG) (NCT01136850) [17]. In the trial, IPTp reduced LBW by 26%, and reduced preterm birth by 38% [17].

## Methods

We recruited women at first prenatal visit in Madang, PNG, between November 2009 and August 2012. The study area is characterised by year-round malaria transmission (*P. falciparum* and *P. vivax*) [18], and LBW is common (17%) [17].

Participants were co-enrolled in a trial that investigated the efficacy of monthly IPTp with azithromycin and sulphadoxine-pyrimethamine (SP), given from second trimester, compared to a single treatment course of SP plus chloroquine at their first prenatal visit, followed by monthly placebo doses (regimen designed to mimic current PNG prevention of malaria in pregnancy policy whilst allowing for participant blinding) [17]. Eligibility criteria included age 16–49 years, singleton pregnancy, and no known co-morbidities. Only trial participants with a dating ultrasound <24 gestational weeks were included [9]. Women with adverse pregnancy outcomes (miscarriage, stillbirth, congenital abnormality) were excluded from analyses.

At enrolment maternal sociodemographic and clinical characteristics were evaluated, maternal anthropometric measurements were taken, and women were screened for anaemia (haemoglobin [Hb] <90 g/L; HemoCue, Sweden). Anaemia was treated with iron/folate supplements and the anthelmintic albendazole, and malaria with quinine (first trimester) or artemether-lumefantrine, according to national guidelines, and insecticide-treated bed nets were provided when available.

A dating ultrasound scan (Logiqbook XP, General Electric Medical Systems, UK) was performed within a week of enrolment using crown-rump length or head circumference (femur length if unavailable) to estimate GA [9]. To monitor fetal growth, women were scheduled for ultrasound scans every six weeks and followed up until birth when birthweight (BW) was measured to the nearest 10 g (Cupid digital scale, Charder Medical, Taiwan). Birth anthropometric measurements were included in analyses only if obtained within 24 hours of delivery. Ultrasound measurements were performed by two clinicians (MO, HWU) and fetal weights (EFW) were estimated from fetal head and abdominal circumference and femur length using standard formulae [19]. A random selection of image stills (10%) was sent for quality control to JW. Women with an abnormal scan were referred to the local obstetrician for further management.

Maternal blood (enrolment, delivery, IPTp visits, passive case detection visits), and placental biopsies were used to characterise malarial infection. Thick smears were used to count the number of asexual parasites per 200 leukocytes (WBC) (or per 500 if <10 parasites/200 WBCs), assuming 8000 WBCs/μL of blood. Slides were declared negative if no parasite was seen in 200 oil-immersion fields at 1000 times magnification following reads by two microscopists. In addition, quantitative real-time polymerase chain reaction (qPCR) was performed on maternal blood [20]. Placental malaria was classified as active (presence of parasitised cells) and past (hemozoin only) [21].

Given the detection of FGR is challenging and not all infants measuring are SGA are growth-restricted we opted to use two indicators of FGR, SGA (cross-sectional assessment) and low fetal weight gain (longitudinal assessment) [13]. Suboptimal fetal growth was suspected upon detection of an EFW or BW below the 10^th^ centile (SGA) of the Hadlock standard (10^th^ centiles of ultrasound-estimated and birthweight-derived standards are similar at term) [19,22], and/or observation of low fetal weight gain, defined as a change in weight z-score (∆z) below the 25^th^ centile of the overall distribution of ∆z, using weight measurements obtained >14 days apart. Factors associated with fetal weight and fetal weight gain were additionally assessed by evaluating differences in continuous outcomes measures, namely weight z-scores and ∆z.

Maternal macronutrient undernutrition was defined as low body mass index (BMI; <18.5 kg/m^2^) or low MUAC (<22 cm) at enrolment, and short stature as maternal height <150 cm [23]. Weekly maternal weight gain was classified as low if in the first quartile for a subset of women with complete maternal weight gain data.

In addition to evaluating the effect of *P. falciparum* and *P. vivax* infection at enrolment (defined as positivity by light microscopy and/or qPCR) we generated weight measurement-specific time-dependent variables of infection to account for time and number of infections in relation to each weight measurement by pooling all available malaria diagnosis data (light microscopy, qPCR, histology) [16]. ‘Recent infections’ were defined as infection during the interval from six weeks prior to the day of fetal weight measurement and ‘any infection’ as infection at any time up until and including the day of weight measurement. Women with active placental malaria were coded as a having had a ‘recent infection’ for the BW measurement, while women with past placental malaria were coded as having had an infection prior to or at enrolment provided they were peripheral blood negative throughout. ‘Recent IPTp’ was defined as IPTp ≤ 6 weeks before a weight measurement.

Statistical analyses were performed using Stata 12.0 (StataCorp, USA). For binary outcomes log-binomial regression models were fitted to calculate risk ratios (RR) and were estimated using generalised estimating equations (GEE) with an exchangeable working correlation structure. Linear mixed effect (LME) models with a restricted maximum likelihood were used to report mean differences in z-scores and ∆z. Both GEE and LME account for repeat measurements of indicators of FGR within individuals. Covariates associated with the outcome measure on univariate analysis (*P* <0.05) were used to generate adjusted RRs and mean differences. Interaction terms for indicators of maternal undernutrition (and gravidity) and malaria were included in log-binomial models to assess the potential effect measure modification of the malaria-SGA relationship by nutritional status and number of previous pregnancies, defined *a priori* as *P* <0.15 of the interaction term.

All participants provided written informed consent. The research was approved by the PNG Institute of Medical Research, the PNG Medical Research Advisory Council and the Melbourne Health Human Research Ethics Committee.

## Results

Of 2793 women enrolled into the parent trial 1863 had no, or late, dating ultrasound scan and 75 were withdrawn for trial reasons, leaving 855 to be enrolled in this ultrasound study. For 122 women pregnancy outcomes were unavailable, 22 suffered a stillbirth, six delivered a congenitally abnormal neonate, and 34 had suboptimal fetal biometric measurements. In a final cohort of 671 participants the mean gestational age (GA) (standard deviation) at enrolment was 19.6 ± 3.7 weeks. Fifty percent were primigravid, most lived rurally, and more than half pursued an income-generating activity (Table 1).

**Table 1:** **Characteristics of pregnant women and risk of measuring small-for-gestational age, Madang, PNG, 2009–2012**

**Characteristic**	**% (N)**		**RR** ^**a**^	**95% CI**	***P***
Maternal age					
16-21	35.9	(241/671)	Referent		
22-26	36.2	(243/671)	0.85	(0.69, 1.03)	
≥27	27.9	(187/671)	0.86	(0.69, 1.07)	0.196
Gravidity					
>1	47.9	(320/671)	Referent		
1	52.1	(351/671)	1.35	(1.13, 1.61)	**0.001**
Ethnicity					
Madang/Morobe	63.5	(426/671)	Referent		
Other	36.5	(245/671)	0.84	(0.70, 1.02)	0.075
Infant sex					
Male	46.3	(307/663)	Referent		
Female	53.7	(356/663)	1.03	(0.86, 1.22)	0.767
Smoking					
No	82.9	(556/671)	Referent		
Yes	17.1	(115/671)	0.98	(0.79, 1.22)	0.886
Betel nut consumption					
No	20.0	(134/669)	Referent		
Yes	80.0	(535/669)	0.88	(0.72, 1.09)	0.238
Literate					
No	8.8	(59/670)	Referent		
Yes	91.2	(611/670)	1.10	(0.81, 1.49)	0.546
Mother generating income					
No	45.5	(298/655)	Referent		
Yes	54.5	(357/655)	1.04	(0.87, 1.24)	0.689
Partner generating income					
No	32.5	(217/667)	Referent		
Yes	67.5	(450/667)	0.87	(0.73, 1.02)	0.086
Area of residence					
Urban	18.5	(124/670)	Referent		
Peri-urban	23.7	(159/670)	1.15	(0.87, 1.52)	
Rural	57.8	(387/670)	1.06	(0.83, 1.37)	0.602
Raised CRP at enrolment (≥5 mg/L)					
No	80.0	(392/490)	Referent		
Yes	20.0	(98/490)	0.89	(0.69, 1.15)	0.367
Gestational week at fetal weight measurement^b^					
22-25	26.3	(395/1,504)	Referent		
26-29	17.2	(258/1,504)	2.24	(1.16, 4.33)	
30-33	11.6	(174/1,504)	4.82	(2.63, 8.81)	
34-37	10.0	(151/1,504)	13.1	(7.47, 22.87)	
≥38	35.0	(526/1,504)	13.9	(8.20, 23.61)	**<0.001**

Note. RR, risk ratio; CI, confidence interval, CRP, C-reactive protein. SGA was defined as a weight below the 10^th^ centile of the Hadlock standard. P-values are for comparison across all groups.

^a^ Unadjusted risk ratio.

^b^ Measurement-specific.

A total of 1504 weight measurements were available for analysis (median per pregnancy 3, range 1–5; EFW = 893, BW = 611). 91.1% (611/671) of BWs were eligible for inclusion: the prevalence of LBW was 15.7% (96/611). Fetuses of 527 women had growth intervals available for analysis (median: 2, range 1–4, total = 833). Most weight measurements were obtained in mid-second and late third trimester (Table 1).

Forty-eight percent of fetuses/newborns (324/671) measured SGA at least once: 275 on one, 43 on two, and six on three occasions, most commonly at delivery (76.9%, 249/324). 37.0% (195/527) of fetuses experienced low weight gain. Ultrasound detection of SGA or low weight gain was strongly associated with LBW, reduced mean birthweights and SGA at birth (Table 2). Primigravidity and GA at weight measurement were associated with measuring SGA (Table 1).

**Table 2:** **Ultrasound detection of SGA and low fetal weight gain, and association with measures at birth**

**Indicator**	**SGA** ^a^	**Not SGA** ^a^	***P***	**Low fetal weight gain** ^b^	**Normal fetal weight gain** ^b^	***P***
LBW	26.5 [18/68]	14.3 [63/441]	**0.011**	25.5 [12/47]	14.1 [31/206]	0.054
SGA at birth	72.1 [49/68]	44.4 [196/441]	**<0.001**	61.7 [29/47]	46.1 [95/206]	0.054
PTB	8.1 [6/74]	4.7 [22/470]	0.215	11.3 [6/53]	2.8 [6/217]	**0.007**
Gestational age (wks)	39.4 (35.1-43.1)	39.4 (25.6-43.7)	0.196	39.1 (35.1, 42.1)	39.4 (34.4, 43.4)	0.083
Birthweight (g)	2718 (2612, 2823)	2961 (2916, 3007)	**<0.001**	2737 (2595, 2880)	2969 (2907, 3031)	**0.002**
Head circumference (cm)	32.5 (32.0, 33.0)	32.9 (32.8, 33.1)	0.101	32.7 (32.1, 33.2)	33.0 (32.7, 33.2)	0.280
Abdominal circumference (cm)	30.9 (30.3, 31.5)	31.8 (31.5, 32.0)	**0.007**	31.3 (30.4, 32.1)	31.8 (31.5, 32.1)	0.191
Crown-heel length (cm)	46.6 (45.8, 47.5)	47.7 (47.4, 48.1)	**0.021**	46.8 (45.8, 47.8)	47.4 (47.0, 47.9)	0.245
Ponderal index (g/[length in cm]^3^)	2.7 (2.6, 2.9)	2.8 (2.7, 2.8)	0.431	2.7 (2.5, 2.9)	2.8 (2.7, 2.9)	0.107
Cord haemoglobin (g/L)	132 (124, 141)	138 (135, 142)	0.178	134 (122, 146)	138.0 (133, 143)	0.500

Note. Values are percent [n], mean (95% confidence interval) or ranks (range). SGA, small-for-gestational-age (<10^th^ percentile of Hadlock standard); *P* <0.05 highlighted in bold.

^a^ Includes 556 women with ≥ 1 ultrasound-estimated fetal weight.

^b^ Includes 275 women with ≥ 1 growth intervals based on EFW only.

Of participants, 13.8%, 21.2%, and 22.8% had a low MUAC, short stature and anaemia (haemoglobin <90 g/L) at first antenatal visit, respectively (Table 3). A low MUAC increased the risk of measuring SGA by 50% in both crude (RR 1.48, 95% CI 1.20, 1.82, P <0.001) and adjusted analyses (aRR 1.51, 95% CI 1.29, 1.76, *P* <0.001) (Table 3). Equally, maternal short stature was a risk factor for measuring SGA (aRR 1.27, 95% CI 1.04, 1.55, *P* = 0.009). Anaemia at enrolment was also associated with an increased risk of SGA (aRR 1.29, 95% CI 1.08, 1.54, *P* = 0.009), and so was low weekly maternal weight gain (aRR 1.21, CI 95% 1.01, 1.45, *P* = 0.042) when assessed in a subset of women. A low enrolment BMI was associated with low fetal weight gain (aRR 2.08, 95% CI 1.62, 2.68, *P* <0.001) (Table 4). Recent receipt of IPTp was associated with increased fetal weight z-scores (adjusted coefficient 0.16, 95% CI 0.04, 0.27, *P* = 0.010) (Table 2), and anaemia with reduced weight ∆z (adjusted ∆z coefficient −0.18, 95% CI −0.34, −0.03, *P* = 0.021) (Tables 3,4).

**Table 3:** **Maternal characteristics and malaria infection, and associations with SGA and fetal weight z-scores**

	**% (N)**	**RR (95% CI) or coefficient [95% CI]**	***P***	**Adjusted RR (95% CI) or coefficient [95% CI]**	***P***
Weight measurements	1,504				
SGA measurements	379				
**Measurement-specific**					
Any malaria infection	21.1 (318/1,504)	1.36 (1.12, 1.65)	**0.002**	1.13 (0.95, 1.34)	0.172
		−0.10 [−0.23, 0.03]	0.115	−0.06 [−0.19, 0.07]	0.372
Recent infection (<6 wks)	10.5 (158/1,504)	1.06 (0.80, 1.41)	0.680	1.03 (0.81, 1.32)	0.799
		0.06 [−0.08, 0.20]	0.403	0.06 [−0.08, 0.21]	0.375
Cumulative infection^a^					
1	18.6 (280/1,504)	1.24 (1.00, 1.52)		1.12 (0.93, 1.34)	
≥2	2.5 (38/1,504)	2.25 (1.63, 3.09)	**<0.001**	1.18 (0.85, 1.63)	0.389
		−0.09 [−0.22, 0.05]		−0.05 [−0.18, 0.09]	
		−0.25 [−0.53, 0.04]	0.155	−0.13 [−0.41, 0.16]	0.581
Recent IPTp	42.4 (637/1,504)	0.22 (0.17, 0.29)	**<0.001**	0.79 (0.57, 1.08)	0.140
		0.20 [0.12, 0.28]	**<0.001**	0.16 [0.04, 0.27]	**0.010**
Low gestational weight gain	23.8 (173/735)	1.15 (0.92, 1.43)	0.237	1.21 (1.01, 1.45)^b^	**0.042**
		−0.16 [−0.35, 0.03]	0.095	−0.18 [−0.37, 0.00]^b^	0.051
**At enrolment**					
Malaria infection	14.9 (100/671)	1.17 (0.92, 1.49)	0.207	1.12 (0.91, 1.36)	0.285
		−0.13 [−0.30, 0.04]	0.137	−0.11 [−0.27, 0.05]	0.186
MUAC <22 cm	13.8 (91/658)	1.48 (1.20, 1.82)	**<0.001**	1.51 (1.29, 1.76)	**<0.001**
		−0.19 [−0.36, −0.02]	**0.031**	−0.18 [−0.34, −0.01]	**0.038**
Body mass index <18.5 kg/m^3^	5.1 (34/664)	1.29 (0.98, 1.69)	0.074	1.33 (0.95, 1.86)	0.103
		−0.19 [−0.36, −0.02]	**0.031**	−0.11 [−0.37, 0.16]	0.436
Height <150 cm	21.2 (141/665)	1.31 (1.10, 1.57)	**0.003**	1.27 (1.04, 1.55)	**0.009**
		−0.19 [−0.36, −0.02]	**0.031**	−0.17 [−0.31, −0.03]	**0.016**
Haemoglobin <90 g/L	22.8 (148/650)	1.12 (0.92, 1.37)	0.266	1.27 (1.06, 1.51)	**0.009**
		0.02 [−0.13, 0.16]	0.840	0.01 [−0.13, 0.15]	0.880

Note. RR, risk ratio, CI, confidence interval, IPTp, intermittent preventive treatment in pregnancy, MUAC, mid-upper arm circumference. *P* <0.05 highlighted in bold. Adjusted analyses included gravidity and gestational age at fetal weight measurement as covariates.

^a^ *P* for comparison across groups.

^b^ Additionally adjusted for gestational age at first maternal weight measurement, time difference between maternal weight measurements.

**Table 4:** **Maternal characteristics and malaria infection, and associations with low fetal weight and change in weight z-scores (∆z)**

	**% (N)**	**RR (95% CI) or coefficient [95% CI]**	***P***	**Adjusted RR (95% CI) or coefficient [95% CI]**	***P***
Number of intervals	833				
Episodes of low fetal weight gain	208				
**Interval-specific**					
Malaria infection before interval	20.7 (172/833)	1.18 (0.87, 1.58)	0.287	1.13 (0.84, 1.52)	0.431
		- 0.12 [−0.30, 0.06]	0.179	−0.11 [−0.29, 0.07]	0.232
Malaria infection during interval	8.2 (68/833)	1.08 (0.72, 1.62)	0.711	0.94 (0.62, 1.44)	0.774
		−0.06 [−0.30, 0.17]	0.607	−0.04 [−0.28, 0.19]	0.721
**At enrolment**					
Malaria infection	13.1 (69/527)	1.18 (0.85, 1.63)	0.324	1.10 (0.79, 1.54)	0.561
		−0.06 [−0.26, 0.14]	0.556	−0.04 [−0.24, 0.16]	0.683
MUAC <22 cm	13.6 (70/516)	1.22 (0.93, 1.59)	0.145	1.12 (0.93, 1.59)	0.156
		−0.18 [−0.36, 0.01]	0.057	−0.16 [−0.34, 0.03]	0.095
Body mass index <18.5 kg/m^3^	4.6 (24/522)	2.10 (1.62, 2.71)	**<0.001**	2.08 (1.62, 2.68)	**<0.001**
		−0.39 [−0.69, −0.08]	**0.014**	−0.35 [−0.66, −0.05]	**0.024**
Height <150 cm	21.4 (115/522)	1.26 (0.97, 1.63)	0.080	1.18 (0.92, 1.53)	0.198
		−0.12 [−0.28, −0.04]	0.150	−0.09 [−0.25, 0.07]	0.256
Haemoglobin <90 g/L	22.3 (115/515)	1.18 (0.91, 1.52)	0.213	1.17 (0.91, 1.51)	0.225
		−0.22 [−0.37, −0.06]	**0.006**	−0.18 [−0.34, −0.03]	**0.021**

Note. IPTp, intermittent preventive treatment in pregnancy; MUAC, mid-upper arm circumference. *P* <0.05 highlighted in bold. Adjusted analyses included gravidity and length of interval as covariates.

A total of 197 *Plasmodium spp.* infections were detected in peripheral blood of 161 women (*P. falciparum* 70.3%, *P. vivax* 29.7%; median number of screens per participant: 4 [range 2–5]). Forty-four percent (86/197) of infections were submicroscopic (detected by qPCR only). Most peripheral infections were detected at enrolment (50.8% [100/197], prevalence 14.9%) and delivery (27.4% [54/197]), and 32 and 44 women had active and past placental infection, respectively.

A history of ‘any infection’ was significantly associated with an increased risk of measuring SGA in an unadjusted model (RR 1.36, 95% CI 1.12, 1.65, *P* = 0.002) but not in a fully adjusted model (RR 1.13, 95% CI 0.95, 1.34, *P* = 0.172) (Table 3). The risk of SGA increased with cumulative infections, but this association did not remain significant in adjusted analyses (Table 3). We did not observe effect measure modification of the malaria-SGA relationship by nutritional status (BMI or MUAC) or primigravidity.

## Discussion

In a cohort of pregnant PNG women maternal undernutrition, short stature and anaemia were associated with indicators of FGR. Malaria infection prior to a weight measurement was associated with an increased risk of SGA in unadjusted but not adjusted analyses. Recent receipt of IPTp was associated with increased fetal weight z-scores.

This is the first study to evaluate causes of FGR in a malaria-endemic region of PNG using ultrasound technology. Strengths of the study include a large sample size, sonographic pregnancy dating and availability of information on potential risk factors for FGR, including malaria.

This research needs to be interpreted in light of its limitations. First, the average number of EFWs measured per fetus was low as a result of higher-than-expected rates of participant non-attendance and limited availability of sonographers, and not all newborns had BWs collected within 24 hours of delivery. This may underestimate SGA prevalence, since fetal growth is thought to be pulsatile and catch-up growth may occur following an insult (e.g. treated malaria infection) [14]. Second, we included BWs in the analysis and defined SGA as any weight below the 10^th^ centile of the Hadlock standard. The use of this nomogram, derived from a Caucasian middle-class cohort, may overestimate SGA amongst differing ethnic groups, as may the inclusion of BWs [24]. Third, despite efforts to closely monitor the malaria infection burden it is possible that infections were missed. Fourth, we used opportunistic sampling of our trial cohort for ultrasound studies, which may have introduced unintentional bias. Fifth, the trial setting (with different antimalarial regimes used) may have resulted in differential effects on parameters of interest. Lastly, not all possible risk factors of FGR and potential confounders of the observed relationships may have been measured and evaluated (e.g. HIV, helminth infection, micronutrient deficiencies).

AC is an important screening tool for FGR [25,26]. We used EFW rather than AC (or a combination of both) in our analyses for several reasons. First, it increases comparability of our findings with other malaria studies [13,16]. Second, many AC measurements were measured postnatally and not by ultrasound; they were not subject to the same stringent QC that ultrasound and birth weight measures were. Third, malaria in early pregnancy and undernutrition have been associated with reduced skeletal growth [11].

Indicators of maternal macronutritent undernutrition were strongly associated with low fetal weight or fetal weight gain. Interventions to improve maternal macronutrient nutritional status including nutritional supplementation merit evaluation in PNG and elsewhere [27]. Anaemia at enrolment was associated with suboptimal fetal weight gain. Anaemia tended to be more common amongst women with concomitant malaria (29.9% vs 21.5%, *P* = 0.070) but nutritional deficiencies and intestinal helminth infections may also contribute to its aetiology. Although it has been proposed that iron supplementation may increase the risk of infection (including malaria) in iron-replete women [28], it is likely that most anaemic women in PNG are iron-deficient, and require supplementation.

We did not observe effect measure modification of the malaria-FGR relationship by maternal nutritional status [16], nor were we able to unequivocally demonstrate a deleterious effect of malaria infection on fetal growth unlike other studies [11]: previous malaria infection was associated with SGA in unadjusted, but not adjusted analyses. This may be because we lack power due to low infection prevalence (reduced further following enrolment by both trial interventions) [17], and comparatively few malaria screening visits and weight measurements, thereby potentially missing infections or episodes of suboptimal fetal growth. Of malaria infections, many were submicroscopic, and their role in causing FGR and LBW remains poorly understood. *P. vivax* caused one third of infections in our cohort. Early *P. vivax* infection has been associated with reduced z-scores and anthropometric measurements [11] [12], but mechanisms are less well understood than for *P. falciparum*, and its deleterious effect on fetal growth may be smaller. Most infections were detected at enrolment. Although there is increasing evidence that malarial infection in early pregnancy can affect fetal growth [29], compensatory processes such as adaptive villous angiogenesis, and catch-up growth, may have mitigated the deleterious effect on fetal growth of some of these infections in the context of early treatment (trial intervention), insecticide-treated bed nets, and close clinical monitoring provided as part of the original trial [13,30]. The risk of SGA secondary to malaria became non-significant in models adjusting for GA at weight measurement. Inclusion of GA as a confounder was necessary because the risk of measuring small increased (linearly) as pregnancy progressed and GA at weight measurement differed between participants. Although we did not corroborate findings of other ultrasound studies, prevention and prompt treatment of malarial infection in pregnancy will reduce LBW and forms an essential part of prenatal care in malaria-endemic areas. Recent receipt of IPTp was associated with increased weight z-scores in this cohort, suggesting it (at least) temporarily prevents FGR through preventing and treating placental malaria or through other, unknown mechanisms that improve fetal growth.

## Conclusions

Over 40% of pregnant women living in a malaria-endemic area in rural PNG had babies that measured SGA in utero or at delivery. Maternal macronutrient undernutrition, short stature and anaemia negatively affected fetal growth, while the low prevalence of malaria may explain its lack of influence on fetal growth. Antenatal interventions to improve nutritional status throughout pregnancy are likely to reduce the risk of FGR and LBW in PNG.
